# Anthropogenic Black Carbon Emission Increase during the Last 150 Years at Coastal Jiangsu, China

**DOI:** 10.1371/journal.pone.0129680

**Published:** 2015-07-22

**Authors:** Kunshan Bao, Ji Shen, Guoping Wang, Chuanyu Gao

**Affiliations:** 1 State Key Laboratory of Lake Science and Environment, Nanjing Institute of Geography and Limnology, Chinese Academy of Sciences, Nanjing, China; 2 Key Laboratory of Wetland Ecology and Environment, Northeast Institute of Geography and Agroecology, Chinese Academy of Sciences, Changchun, China; Griffith University, AUSTRALIA

## Abstract

Black carbon (BC) is one of the major drivers of climate change and a useful indicator of environmental pollution from industrialization, and thus it is essential to reconstruct the historical trend in BC flux to better understand its impact. The Yancheng coastal wetland reserve in Jiangsu province is an area sensitive to global sea level change and is also located in the most developed as well as most polluted region of China. We investigated the concentration and historical flux of BC over the past 150 years through geochemical analysis of two ^210^Pb-dated sediment cores from Yancheng coastal wetland. Measured BC contents ranged from 0.24 mg g^-1^ to 1.41 mg g^-1^ with average values of 0.51mg g^-1^-0.69 mg g^-1^, and BC fluxes ranged from 0.69 g m^-2^ yr^-1^ to 11.80 g m^-2^ yr^-1^ with averages of 2.94g m^-2^ yr^-1^-3.79 g m^-2^ yr^-1^. These values are consistent with other records worldwide. Both BC content and flux show a gradual and continuous increase over time and clearly reflect increased emissions from anthropogenic activities. The BC records have a significant peak in recent years (from 2000 to 2007), which is accompanied by the sharp increase of energy consumption and total carbon emission in the region. It is reasonable to conclude that changes in BC from increasing human activities have controlled BC fluxes during the last 150 years. Industrial contamination, especially BC emission, in the coastal region of eastern China should be taken into account when developing management strategies for protecting the natural environment.

## Introduction

Black carbon (BC) is a molecularly diverse organic product of incomplete combustion of biomass and fossil fuels and is one of the major components of atmospheric aerosols [1]. It plays a critical role in climate change because of its efficient light-absorbing character [2] and has been identified as the second largest contributor, after CO_2_, to anthropogenic radiative forcing [3]. Globally, BC aerosols derived from burning vegetation, are emitted to the atmosphere at a rate of 50–270 Tg C annually [4]. Emissions of BC from fossil fuel use has been increasing almost linearly, from about 1.0 Tg C in 1850 to 2.2 Tg C in 1900, 3.0 Tg C in 1950 and 4.4 Tg C in 2000 [5]. As one of the major source regions of BC aerosol, BC emissions in China accounts for roughly 25% of the global anthropogenic production [6,7]. As a result, BC is widespread in the environment including in soil and sediment. BC in soils and sediments exists as a carbonaceous substance of pyrogenic origin that is relatively resistant to biological and chemical degradation [8,9]. Therefore, BC represents a C sink in essence with a long residence time (up to 2400 years in soils) [10]. Owing to the above two reasons (being direct radiative forcing and potential C sink), there has been an escalation in interest in BC [11–19].

Coastal wetlands represent one of the most significant ecosystems in the world providing high resilience against extreme weather, wide varieties of plant and animal species, and significant social-economic benefits to fisheries [20]. Coastal wetlands play an important role in the global C cycle [21–23], but are sensitive to global climate change. The potential for soil C storage, including in coastal wetland soils, is enormous with a global potential for C sequestration through changes in soil management practice estimated to be 1.2–2.6 Pg C per year [24]. The key factors controlling vertical marsh accretion and associated C sequestration are the addition of mineral sediments as well as the contribution of soil organic matter [23]. However, coastal wetlands are vulnerable to climate change and direct anthropogenic disturbance. As a result of continued population growth and increasing global food demand, around 25–50% of the world’s coastal wetlands have been converted into farmland and aquaculture land over the last 50–100 years [25,26].

In China, meeting future food and energy demands while mitigating potential detrimental environmental impacts has emerged as one of the country’s greatest challenges, particularly in relation to the exploitation and conservation of coastal wetlands [26]. For example, the greatest expanse of coastal wetlands in China isin north Jiangsu province and these provide habitat for many rare and endangered species. Although two wetland reserves of international importance have been established, the Dafeng National Nature Reserve (Ramsar site no. 1145) and the Yancheng National Nature Reserve (YNNR, no. 1156), the outlook for broader protection is not optimistic. From 1988 to 2006, grass flats in YNNR have decreased by about 900 ha yr^-1^, while farmland and pond area have increased by 600 ha yr^-1^ and 1400 ha yr^-1^, respectively [27]. This is not an isolated case in China; it has been reported that 57% of China’s coastal wetlands have disappeared due to land reclamation since the 1950s [28].

We present here the first sequence of BC deposition flux over the past 150 years in coastal wetland sediments of north Jiangsu, China. Our aims are the reconstruction of historical trends of atmospheric BC emissions and the linking of this record with the development of industry, transport and agricultural activities. This study will be helpful in revealing anthropogenic effects on the regional environment and providing support for policy-making decisions for sustainable development.

## Materials and Methods

### Ethics Statement

All necessary permits for field sampling were obtained from Jiangsu Yancheng Wetland National Nature Reserve, Rare Birds. The study area is located in the experimental area of the Yancheng National Nature Reserve (YNNR, Ramsar site no. 1156), and the field studies did not involve endangered or protected species.

### Study Area

The coastal region in Yancheng of Jiangsu province faces the Yellow Sea to the east and the Yangtze River to the south. Total area of the Yancheng wetlands is 4,530 km^2^ (ca. 30% of the municipality’s total area) and stretch for about 580 km along the coast, accounting for 70% of the provincial total and 14.3% of the national total. The wetlands consist primarily of extensive inter-tidal mudflats, tidal creeks and river channels, salt marshes, reed beds, and marshy grasslands that provide desirable habitats for numerous species of flora and fauna of global and national importance. Moreover, the wetlands provide important ecosystem services to local communities, such as improvement of water quality by assimilating household and industrial wastes that are rapidly increasing in Yancheng municipality. The Yancheng wetlands have been listed in the world network of biosphere conservation (WNBP) by the United Nations in 1992, and have become the hotspot of wetland research for their significance. However, the Yancheng coastal wetlands have been experiencing rapid degradation due to the rapid economic development as well as frequent land use changes. Land use along this coastal region includes agriculture farming, aquaculture, and solar salt production. In recent times, harbor building, wind power generation, and tourism activities have increased, along with the associated sewage and solid wastes production [29].

**Fig 1:**
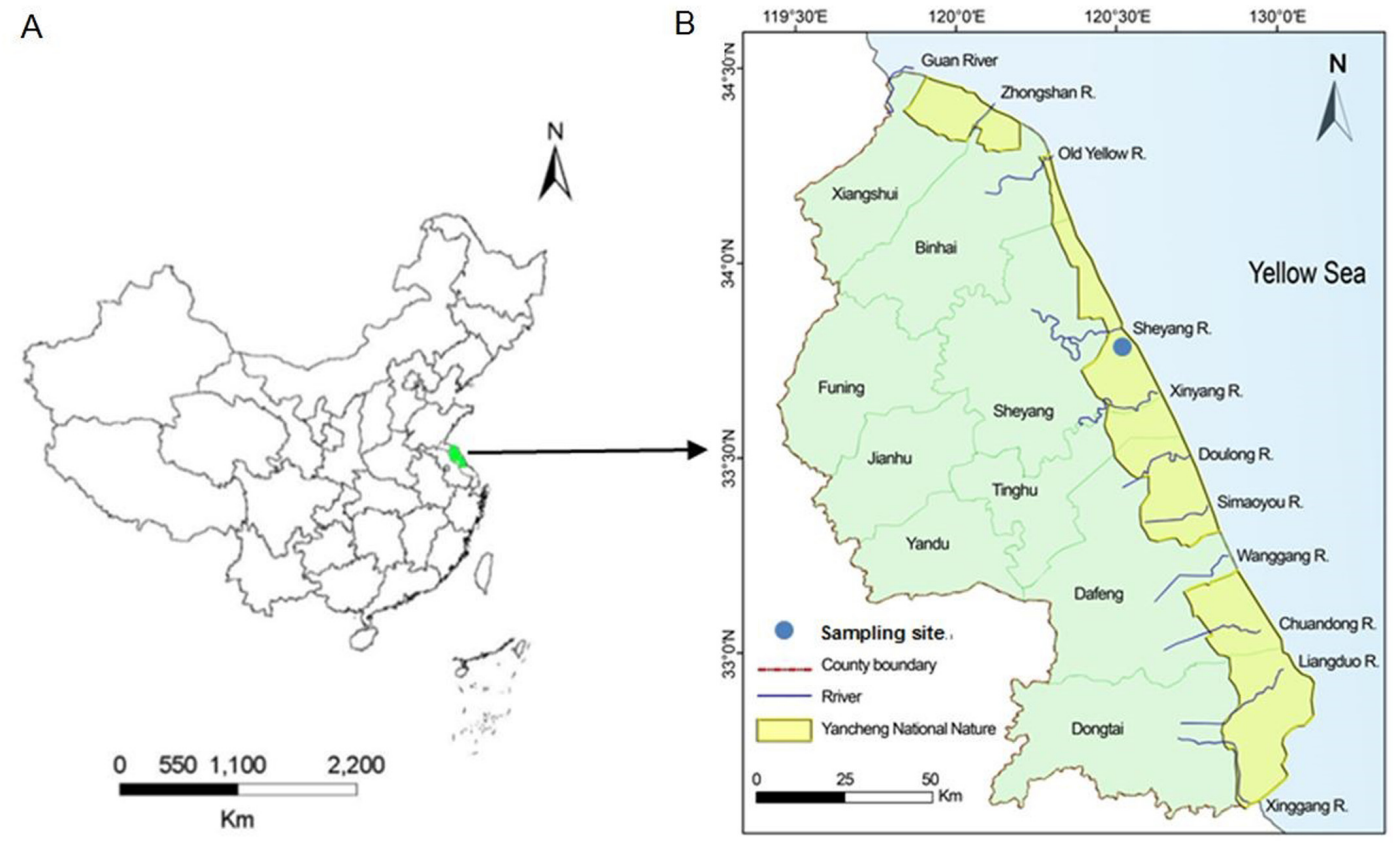
Location of sampling site. **(A)** China map showing the location of Yancheng city in Jiangsu province. **(B)** Yancheng map showing the National Nature Reserve area and the sampling site.

The sampling site is situated in Sheyanggang of Yancheng (Fig 1), which is a typical tidal ecosystem and very close to Xingyanggang in the same region. This wetland is representative of the north subtropical zone with average precipitation of 1010 mm yr^-1^ and annual average temperature of 14.4°C. The tidal flat is affected by the marine monsoon climate with prevailing southeastern winds in summer and prevailing northwestern winds controlled by tropical depression in winter [30]. This site has a plain sedimentary geomorphology (average slope: 0.055%) formed by fluvial and coastal sedimentary processes since the Late Pleistocene. The soils are classified into Anthrosols, Fluvisols, and Cambisols according to the formation process [31]. At present, the wetland landscape consists of bare silt-sand mixed flat, *Spartina alterniflora* flats, *Suaeda salsa* falts, and *Phragmites australis* flats as one progress from the sea inland [32]. The *Spartina alterniflora* flat and the *Suaeda salsa* flat usually stagger and overlap in their distributions, but the former is one of the dominant species, with a stronger root system and a greater tolerance to salinity and submergence. As a native plant zone, the *Phragmites australis* flat is mostly accompanied with the paddy and aquafarm fields, and their area is decreasing due to serious anthropogenic disturbance.

### Core-sampling and Slice-sectioning

**Fig 2:**
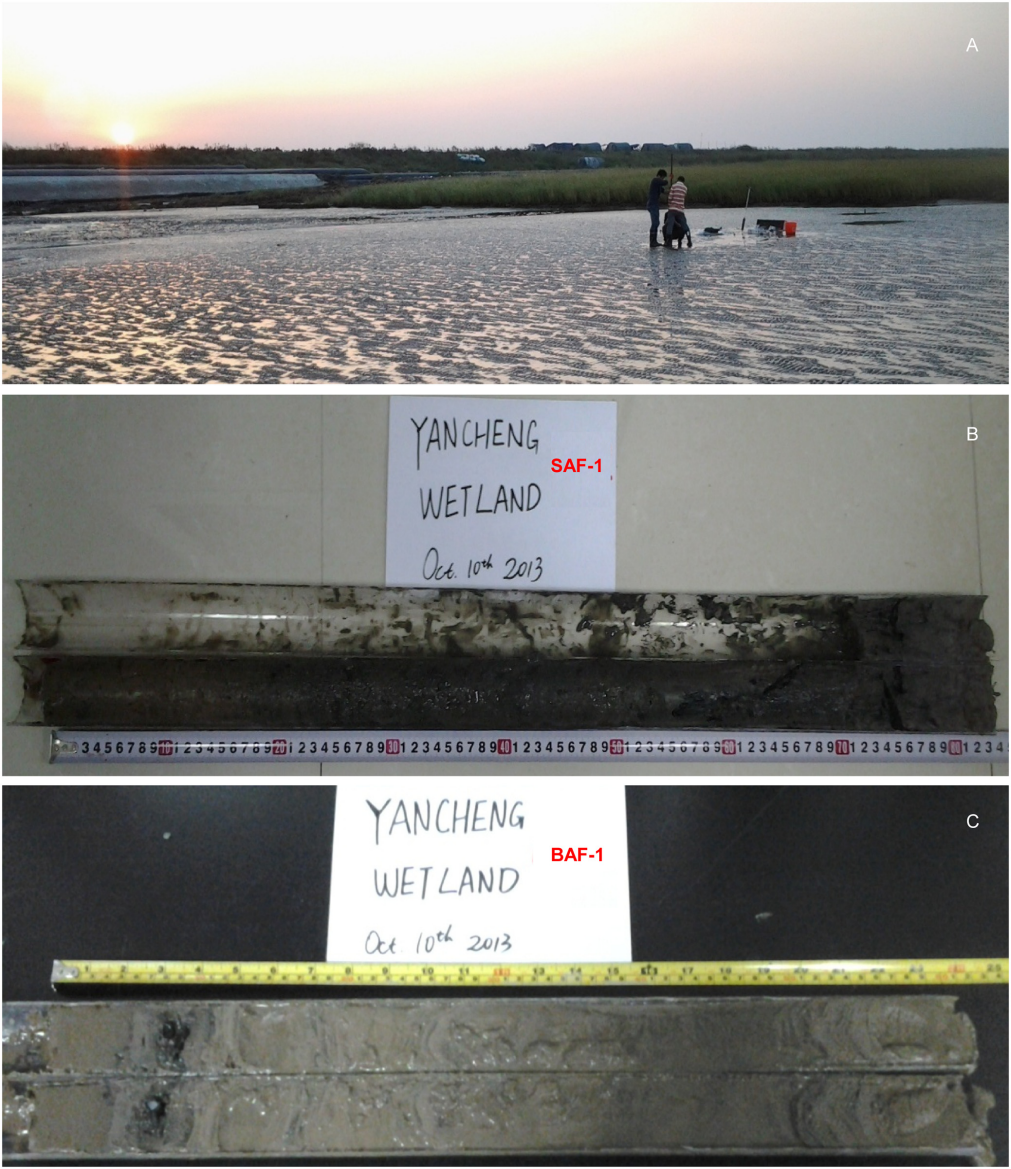
Photos of core sampling. **(A)** A photo showing the sampling site scene at Yancheng coastal wetland and core sampling in the field. **(B)** and **(C)** Photos showing the sediment cores of SAF-1 and BAF-1. PVC pipes were cut in half to show the lithologic character in the laboratory.

Two sediment cores were collected from representative habitat zones using a gasoline-powered corer (Eijkelkamp, Netherlands) in October 2013, a *Spartina alterniflora* flat (labeled SAF-1, N 33°46′34″, E 120°31′49″, Altitude 3 m) and a bare flat (labeled BAF-1, N 33°44′37″, E 120°31′50″, Altitude 1 m) (Fig 2). The latitude, longitude, and altitude of both sampling sites were determined with a portable global positioning system (Garmin GPS 62SC, Garmin International, Olathe, KS). The cores were taken back to the laboratory, split in half length-wise, with one-half frozen for later use, and the other half cut into 1 cm slices using a stainless steel semicircle blade with half pipe-diameter size. All slices were packed into labeled zip-lock polyethylene plastic bags for storage and further preparation.

### Physical and Chemical Analysis

Water content (%) and dry bulk density (g cm^−3^) of samples were determined by weighing a volumetric sub-sample of each slice of the sediment cores before and after freezer drying overnight. Mass magnetic susceptibility was quantified from the homogenized, dried samples using a Bartington Instruments MS2 sensor.

### Determination of Age Using ^210^Pb and ^137^Cs

Bulk weighed dry samples were sealed in plastic test tubes with caps for ^210^Pb dating by gamma spectrometry using a well-type coaxial low background intrinsic germanium detector (Ortec HP Ge GWL series, Oak Ridge, TN, USA). Radioactivity levels of ^210^Pb were determined via gamma emissions at 46.5 keV. Emissions of^226^Ra with the 295 keV and 352 keV (from the daughter nuclide ^214^Pb) were determined after 3 weeks of storage in sealed containers to reach radioactive equilibrium. Radioactivity of ^137^Cs was measured using the 662 keV photo peak. Standard sources and sediment samples of known activity were provided by the China Institute of Atomic Energy and used to calibrate the absolute efficiencies of the detectors. Counting times were typically in the range 50,000–86,000 s, giving a measurement precision of between ±5% and ±10% at the 95% level of confidence, respectively. Supported^210^Pb in each sample was assumed to be in equilibrium with the in-situ ^226^Ra, and unsupported ^210^Pb activities were determined from the difference between the total ^210^Pb and the supported ^210^Pb activity.

### Black Carbon Analysis

Black carbon in the sediments was analyzed by the dichromate oxidation method according to [19,33]. The predetermined amount (1 g) of a sample was digested for 20 h in 10 mL HCl (1 mol L^-1^) in plastic centrifuge bottles. The contents were centrifuged and the residue was added to a 10 mL mixture (v/v, 1/2) of HCl (3 mol L^-1^) and HF (22 mol L^-1^) for 20 h. Then the samples were centrifuged again and the residue was soaked in 10 mL HCl (1 mol L^-1^) for 10 h. This is the first step to remove inorganic carbon; after that, the residue consists of organic matter, kerogen and BC. The second step was to remove NPOC (non-pyrogenic organic carbon) in residues. We used 30 mL NaOH (0.1 mol L^-1^, 12 h, twice) to remove humic acid and a mixed solution of K_2_Cr_2_O_7_ (0.1 mol L^-1^) and H_2_SO_4_ (2 mol L^-1^) (60 h, and keep mixture stay yellow) to remove kerogen. All steps were treated in 55°C bath [34]. The residual carbon (as BC) was quantified using a continuous-flow isotope ratio mass spectrometer (CF-IRMS) at the Northeast Institute of Geography and Agroecology, Chinese Academy of Sciences. The CF-IRMS system consists of an EA (Flash 2000 series) coupled to a Finnigan MAT 253 mass spectrometer. The combustion temperature was set at 960°C. Standard samples with known carbon contents (IRMS certified reference: BN/132357) were used to calibrate the measurement and to monitor the working conditions. The content of BC in wood char [35] tested here (48.7% -50.2%, n = 3) was consistent with previous reports (48.4% -55.8%, n = 4) using the dichromate oxidation method in different laboratories [10].

### Calculation of SR, MAR and BC Flux

Due to tidal and anthropogenic effects, which results in varying sedimentation rates, the core chronology was determined using the constant rate of supply (CRS) dating model [36] according to Eq 1. Sediment rate (SR, cm yr^-1^) was calculated based on the ^210^Pb inferred chronologies according to Eq 2 [37]. To estimate inventories and burial fluxes of sediment mass and BC components in the sediment cores of a given area, several sediment properties including DBD, SR and sediment porosity (SP, dimensionless) must be taken into account in addition to the sediment mass and the BC concentration [38]. SP was defined as one minus the ratio of DBD and the solid-grain density (SGD) which was taken as 2.7 g cm^-3^ [39] according to Eq 3. Mass accumulation rate (MAR, g cm^-2^ yr^-1^) and the BC burial flux (g m^-2^ yr^-1^) were estimated using Eqs 4 and 5.

$$T_{Z}(yr)=-\frac{1}{\lambda }\times Ln\frac{I_{Z}}{I_{tot}}$$ (1)

$$SR_{Z}(cm^{}yr^{-1})=\frac{Z(cm)}{T_{Z}(yr)}$$ (2)

$$SP=1-\frac{DBD}{SGD}$$ (3)

$$MAR(g^{}cm^{-2}{}^{}yr^{-1})=DBD(g^{}cm^{-3})\times SR_{Z}(cm^{}yr^{-1})\times (1-SP)$$ (4)

$$BC^{}Flux(g^{}m^{-2}{}^{}yr^{-1})=BC(mg^{}g^{-1})\times MAR(g_{}cm^{-2}{}^{}yr^{-1})\times 10$$ (5)

Where T_Z_ is the age of layer at depth Z (cm), I_Z_ and I_tot_ refer to the inventory of unsupported ^210^Pb at depth Z (cm) and the total inventory of unsupported ^210^Pb in the core section (both are calculated by direct numerical integration), λ is the ^210^Pb decay constant (0.0311 yr^-1^), and 10 in Eq 5 is a unit conversion factor.

### Data Statistical Analysis

Values of mean, standard deviation, minimum and maximum values were calculated for core variables. Regression analysis was performed to examine the changing pattern of BC content and flux with time. A statistical significance was determined at the *P* = 0.05 level except if indicated differently. These procedures were performed using the SPSS 11.5 software package [40].

## Results and Discussion

### Physicochemical Properties of Sediment

**Fig 3:**
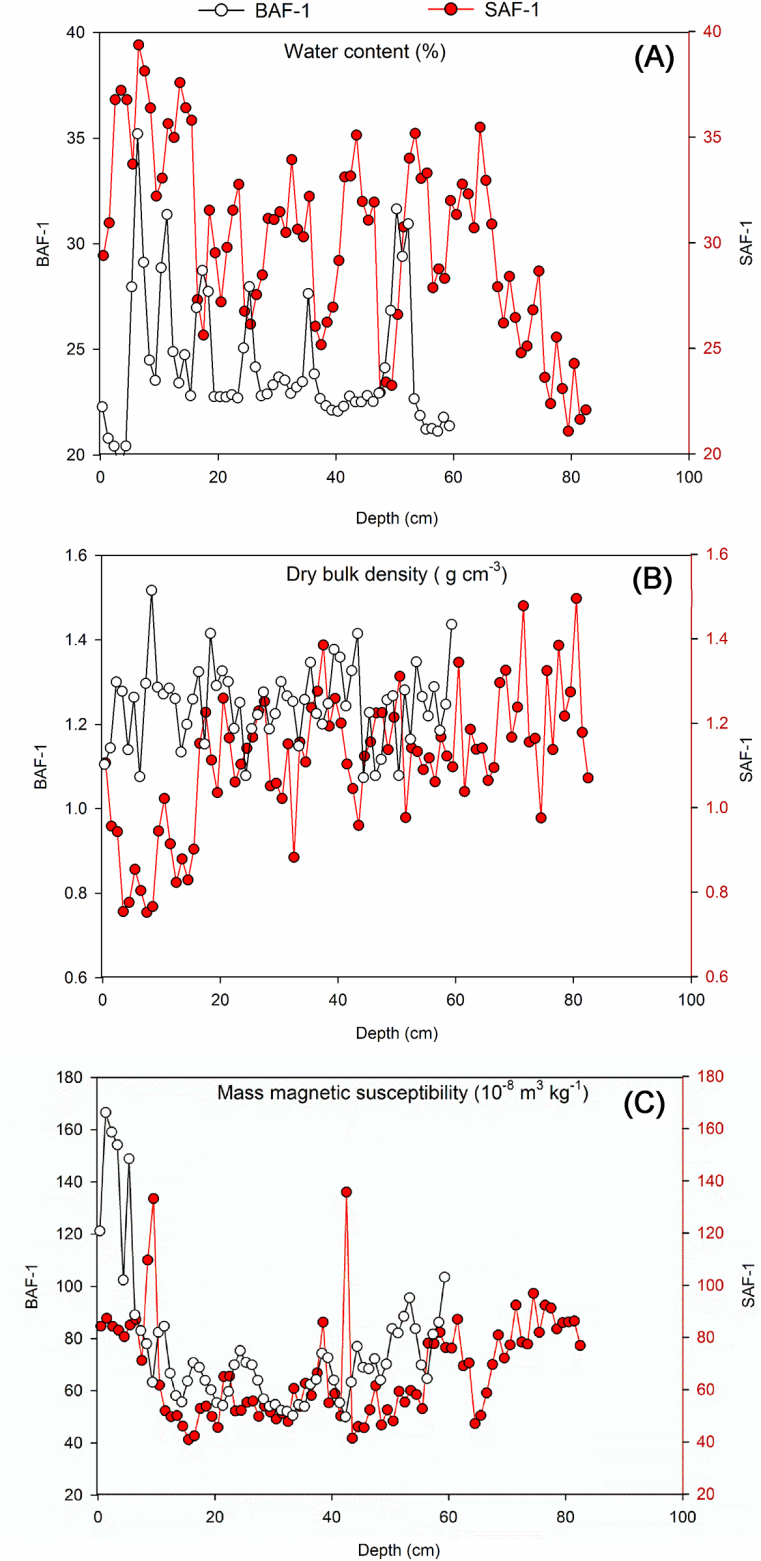
Depth variations of physicochemical parameters of two sediment cores (SAF-1 and BAF-1) in Yancheng coastal wetland, China. **(A)** Water content (%). **(B)** Dry bulk density (g cm^-3^). **(C)** Mass magnetic susceptibility (10^−8^ m^3^ kg^-1^).

Water content of both SAF-1 and BAF-1 cores ranged from 20% to 40%. The distributions were similar for the two cores and characterized by the highest value occurring at the subsurface sections, with a general decrease on moving to the deeper layers (Fig 3A). This is consistent with a previous report of water content for the bare silt zone (average 30%, the whole core of 120 cm) and for the *Spartina alterniflora* zone (average 40%, the lower section of 20–120 cm and significantly elevated in the surface 20 cm) in this same coastal wetland [32]. However, a peak-valley pattern of water content with depth for both cores was observed here, which is different from the above-mentioned projects, probably reflecting the periodic variation in ground water level that is mainly affected by the sea water level. Dry bulk density of the SAF-1 core ranged from 0.75 g cm^-3^ to 1.45 g cm^-3^, and that of the BAF-1 core ranged from 1.07 g cm^-3^ to 1.52 g cm^-3^. It was obvious that DBD of SAF-1 increased with depth (Fig 3B), while the pattern of both cores below ca. 20 cm was consistent. Lower DBD values of SAF-1 than BAF-1 for the topmost sections were likely a result of the existence of plant matter in SAF-1; the top layer of the sediment in SAF-1 was black and contained a large content of decayed *Spartina alterniflora*. Variations in mass magnetic susceptibility for the two cores were quite similar and were elevated in the upper layers of the profiles (Fig 3C). Magnetic susceptibility is directly linked in this case to a concentration of anthropogenic ferromagnetic particles and is dominated by high values of magnetite that are produced during the combustion of fossil fuel [41]. As a result, the pattern of mass magnetic susceptibility enabled us to define the area which was severely contaminated by industrial activities.

### Radioisotope Chronology and Sediment Rate

**Fig 4:**
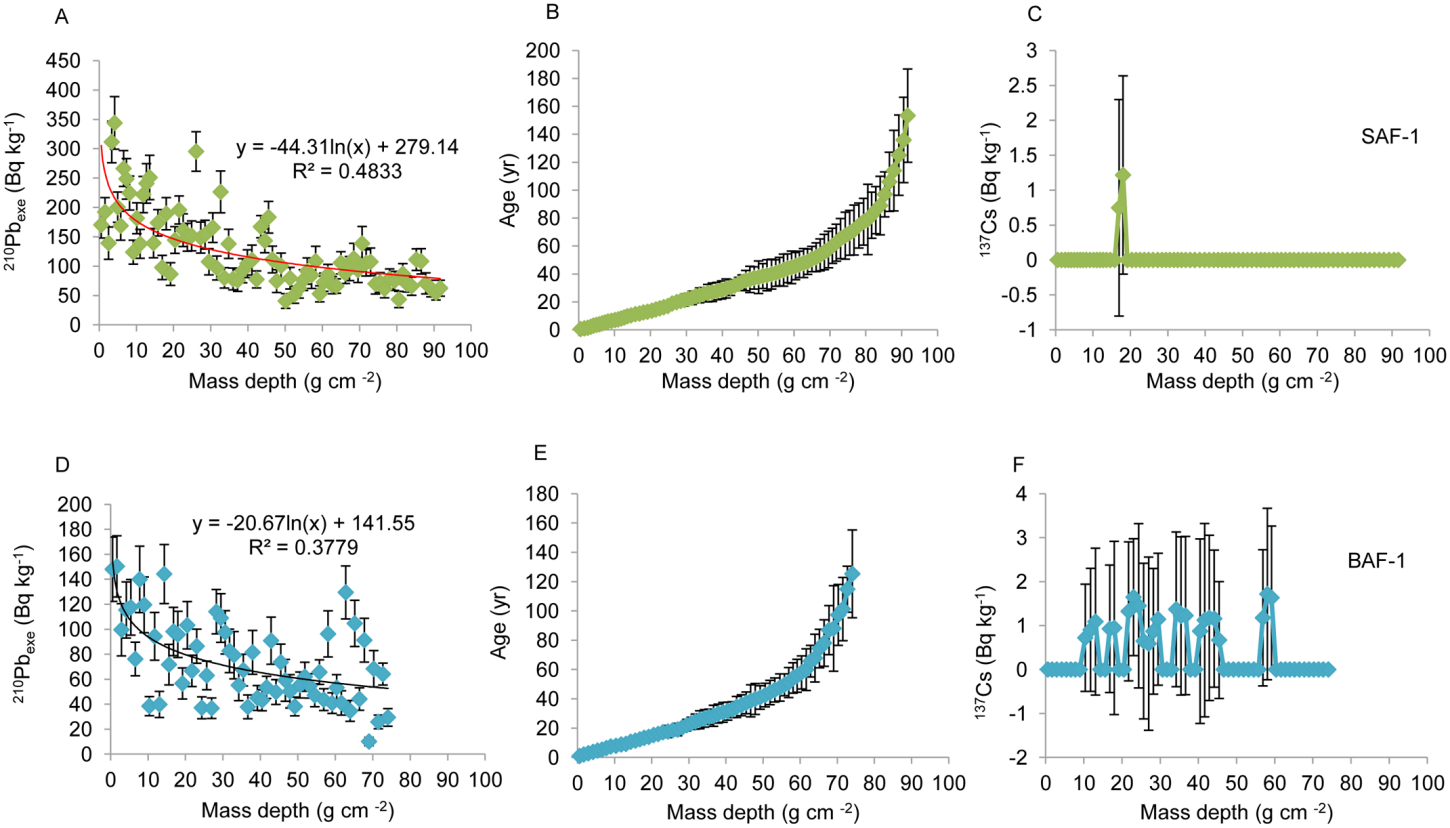
Radioisotope results for ^210^Pb and ^137^Cs. **(A)** ^210^Pb activity (Bq kg^-1^), **(B)** ^210^Pb-inferred chronologies (yr) and **(C)** ^137^Cs activity (Bq kg^-1^) plotted against mass depth (g cm^-2^) for SAF-1 core. **(D)** ^210^Pb activity, **(E)** ^210^Pb-inferred chronologies and **(F)** ^137^Cs activity plotted against mass depth for BAF-1 core. Error bars represent 1 standard deviation (SD) from counting uncertainty.

**Fig 5:**
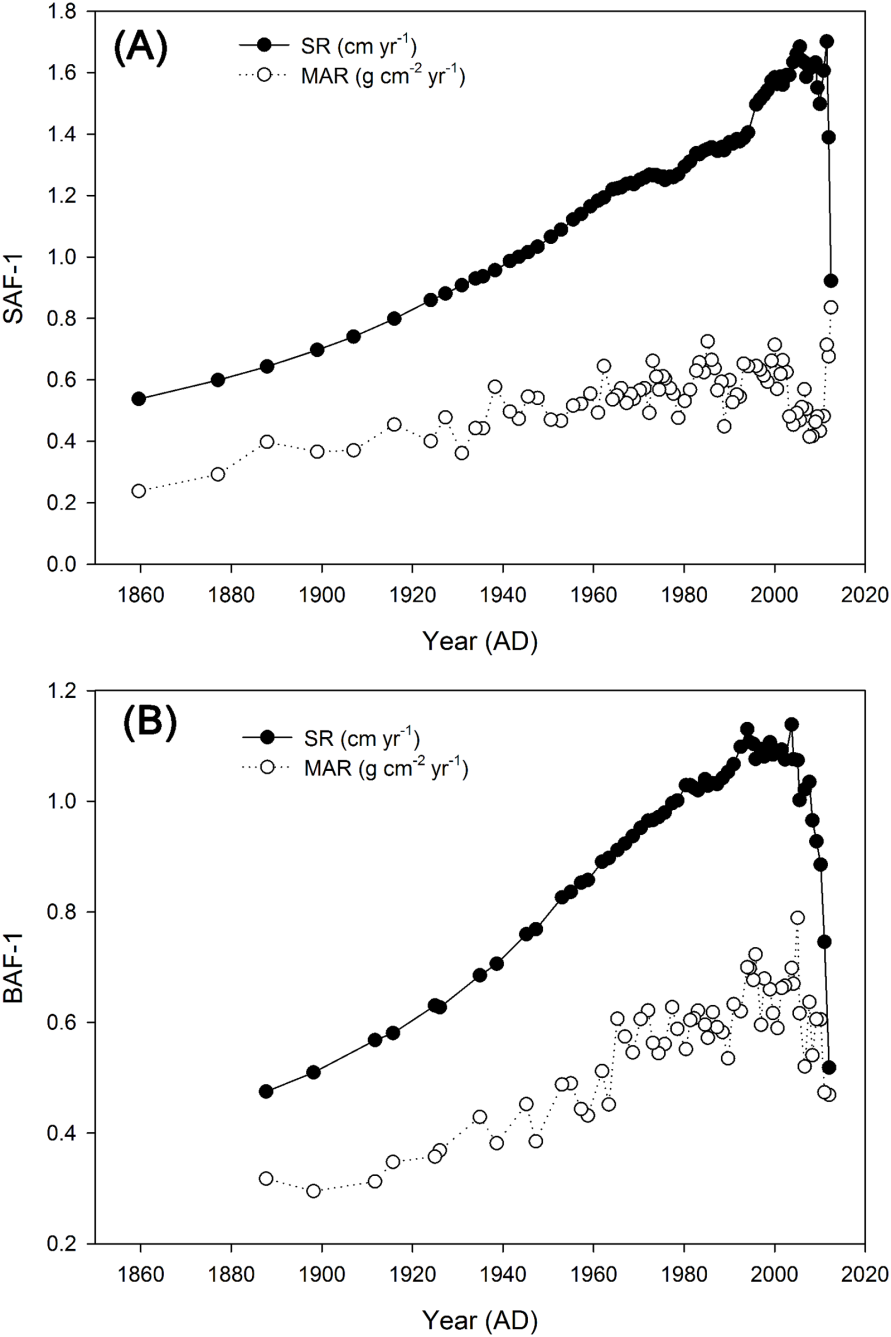
Sediment rate (SR, cm yr^-1^) and mass accumulation rate (MAR, g cm^-2^ yr^-1^) plotted against age determined from ^210^Pb (calendar year AD) of two sediment cores (SAF-1 and BAF-1) in Yancheng coastal wetland, China.

Radioisotope results for ^210^Pb were plotted and are shown in Fig 4A and 4D for the two cores. Their unsupported ^210^Pb activities (excess ^210^Pb, ^210^Pb_exe_) presented a relatively well-defined logarithmic decrease with depth, and they became negligible at the bottom of both cores because the total ^210^Pb is in equilibrium with ^226^Ra (^214^Pb). The continuous ages were calculated using the CRS dating model and the age-depth models for both cores were plotted (Fig 4B and 4E). The sediment records cover about 150 years for SAF-1 and 125 years for BAF-1, extending back to AD 1860 and AD 1885, respectively. The ^137^Cs activity for both cores was relatively low along the entire profiles (Fig 4C and 4F). The maximum activities of ^137^Cs were 1.22 Bq kg^-1^ at a depth of 20 cm (i.e., 18 g cm^-2^) for SAF-1 and 1.72 Bq kg^-1^ at a depth of 46 cm (i.e., 58 g cm^-2^) for BAF-1, which is consistent with the peak value of ^137^Cs (1.53 Bq kg^-1^) in the same wetland [30]. The substantial difference in depth for the maximum ^137^Cs activities in the two cores indicates that the ^137^Cs technique did not provide a reliable chronostratigraphic index in our sediment samples. This is likely because ^137^Cs was not well preserved in silt-fine sand and mud-sand sediments of Yancheng coastal wetland [30]. There exists a variable adsorption characteristic of different grain size material, as shown by the variable sedimentology (Fig 2C). Consequently, the results of ^137^Cs activities were not used to check the date in this study. Using the ^210^Pb-derived age, the calculated SR of the SAF-1 core ranged from 0.54 cm yr^-1^ to 1.70 cm yr^-1^, with an average of 1.28 cm yr^-1^. The BAF-1 core ranged from 0.47 cm yr^-1^ to 1.14 cm yr^-1^, with an average of 0.93 cm yr^-1^. MAR of both cores ranged from 0.24 g cm^-2^ yr^-1^ to 0.84 g cm^-2^ yr^-1^ and averaged 0.55 g cm^-2^ yr^-1^. The average SR is approximately consistent with the rate of 1.2 cm yr^-1^found on the Xingyanggang tidal flat (only 15 km in distance from our study site) [30] but smaller than the rate of 3.0 cm yr^-1^found on the Wanggang salt marsh (about 75 km southeast of our study site) [42]. The overall patterns were quite similar for both cores, with a general increase over time reaching a maximum value around 2007 AD and then a decrease afterwards (Fig 5). This dropping period of SR corresponded to the uppermost 5 cm section for both cores, which would be possibly affected by the intensive hydrodynamic force that resulted in less effective deposit.

### Black Carbon Concentration and Flux

**Fig 6:**
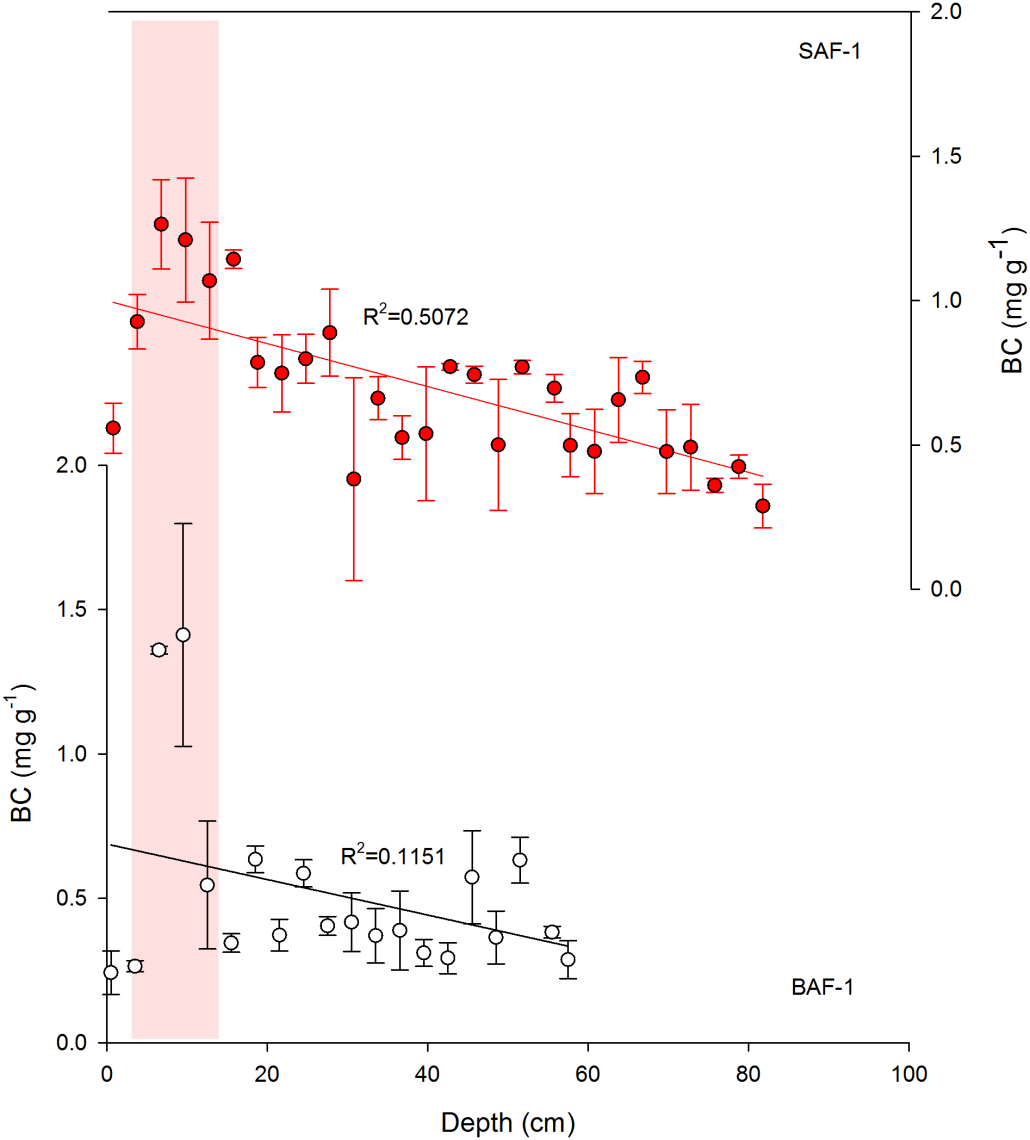
Depth variations of black carbon (BC, mg g^-1^) of two sediment cores (SAF-1 and BAF-1) in Yancheng coastal wetland, China. An obvious peaking section of BC content is marked by red bar (the surface 15 cm). Error bars represent the standard deviation (SD) of the mean of three parallel samples.

The measured BC contents ranged from 0.29 mg g^-1^ to 1.26 mg g^-1^ for SAF-1 and from 0.24 mg g^-1^ to 1.41 mg g^-1^ for the BAF-1 profile. The average values of both cores were 0.69 mg g^-1^ and 0.51 mg g^-1^, respectively. BC concentrations for the two cores showed significantly increasing trends upward (R^2^ = 0.5075 for SAF-1 and 0.1151 for BAF-1), peaking in the near surface layers (around 5–15 cm) (Fig 6). In the uppermost 5 cm sections, there was a slight decline in BC content, which is probably because BC concentration may be strongly affected by dilution with detrital matter and varying water contents [17,43,44]. To properly account for differences in density and water contents, BC concentrations were converted to BC deposition fluxes to better reflect the real variation of BC input. BC fluxes of the SAF-1 core ranged from 0.79 g m^-2^ yr^-1^ to 7.58 g m^-2^ yr^-1^, with an average of 3.79 g m^-2^ yr^-1^; that of the BAF-1 core ranged from 0.69 g m^-2^ yr^-1^ to 11.80 g m^-2^ yr^-1^, with an average of 2.94 g m^-2^ yr^-1^.

Current concerns about the changing carbon cycle of the coastal ocean and global carbon balance [45] has stimulated a growing interest in documenting coastal environmental and climatic changes over recent time periods, especially the last few hundred years. Relevant studies on short-term BC accumulation worldwide are available to ensure comparisons of BC content and flux in different geological records estimated by different methods (Table 1). Our results are comparable to most studies, and quite similar to those for Taihu Lake (about 250 km away) in Jiangsu province [46]. The consistency of these values suggests that BC content and flux records in Yancheng coastal wetland could reflect the dynamic variations of atmospheric BC aerosols in this region.

**Table 1:** Comparisons of averages of black carbon concentrations (mg g^-1^) and deposition fluxes (g m^-2^ yr^-1^) in a world context.

Sediment type	Region	Age span	Concentration	Flux	Method	Reference
Wetland sediment	Yancheng coastal wetland, China	1860–2013	0.51–0.69	2.94–3.79	Acid Dichromate Oxidation	This study
	Sanjiang Plain freshwater wetland, China	1850–2010	3.2–61.2	4–112	Acid Dichromate Oxidation	[19]
	Pearl River estuary, China	1900–2008	0.4–4.6	6.9–25.6	Cr_2_O_7_	[51]
Lake sediment	Nam Co, Tibetan Plateau, China	1857–2009	0.49–1.09	0.12–0.44	TOR-IMPROVE	[17]
	Dahai, North China	1800–2001	0.52–4.90	0.6–7	TOR-IMPROVE	[46]
	Taihu, East China	1825–2003	0.43–1.95	1.15–6.89	TOR-IMPROVE	[46]
	Chaohu, China	1850–2010	0.05–0.42	NA	CTO375	[16]
	West Pine Pond, New York State, USA	1835–2005	0.6–8	0.026–0.77	TOT-STN	[44]
	Ledvica, Alps, Slovenia	1815–1998	3.6–9.2	0.3–1.3	CTO-375	[52]
	Engstlen, Alps, Switzerland	1963–2008	1.5–3.3	2.1–7.4	CTO-375	[53]
	StoraFrillingen, Aspvreten, Sweden	1000s-2005		0.05–0.40	CTO-375	[43]
	Amazon, Brazilian	1978–1996	1–27	3–90	Acid Dichromate Oxidation	[54]
Ocean sediment	Palos Verdes Shelf, CA, USA	1886–1996	1.2	7–10	CTO375	[55]
	New England Harbors	1886–1996	2–7	5–23	CTO375	[55]
	Deep sea sediment, South Atlantic Ocean	1860–2000	0.4–1.7	0.005–0.078	CTO375	[56]
	Swedish continental shelf	SS	0.58–17.66	2.67–80.67	CTO375	[38]
	Mud area in Bohai Sea	1910–2006	0.24–0.49	0.26–0.53	CTO375	[57]
	Bohai Bay intertidal, China	SS	0.22–0.92	NA	CTO375	[58]
	Bohai Bay, China	SS	1.85–2.45	NA	CTO375	[59]
	North Yellow Sea	SS	0.10–0.93	NA	CTO375	[59]
	Jiaozhou Bay, China	SS	0.10–0.97	NA	CTO375	[59]
	South China Sea	SS	0.10–1.86	NA	CTO375	[59]
	East China Sea	SS	0.02–0.14	NA	CTO375	[60]
	East China Sea Inner continental shelf	SS	0.21–0.88	NA	CTO375	[61]

TOR-IMPROVE, thermal optical reflectance, following IMPROVE-A protocol. TOT-STN, thermal optical transmittance, following STN protocol. CTO-375, chemo-thermal oxidation at 375°C. SS, surface sediment. NA, not available.

### Historical Trend of Black Carbon Flux

**Fig 7:**
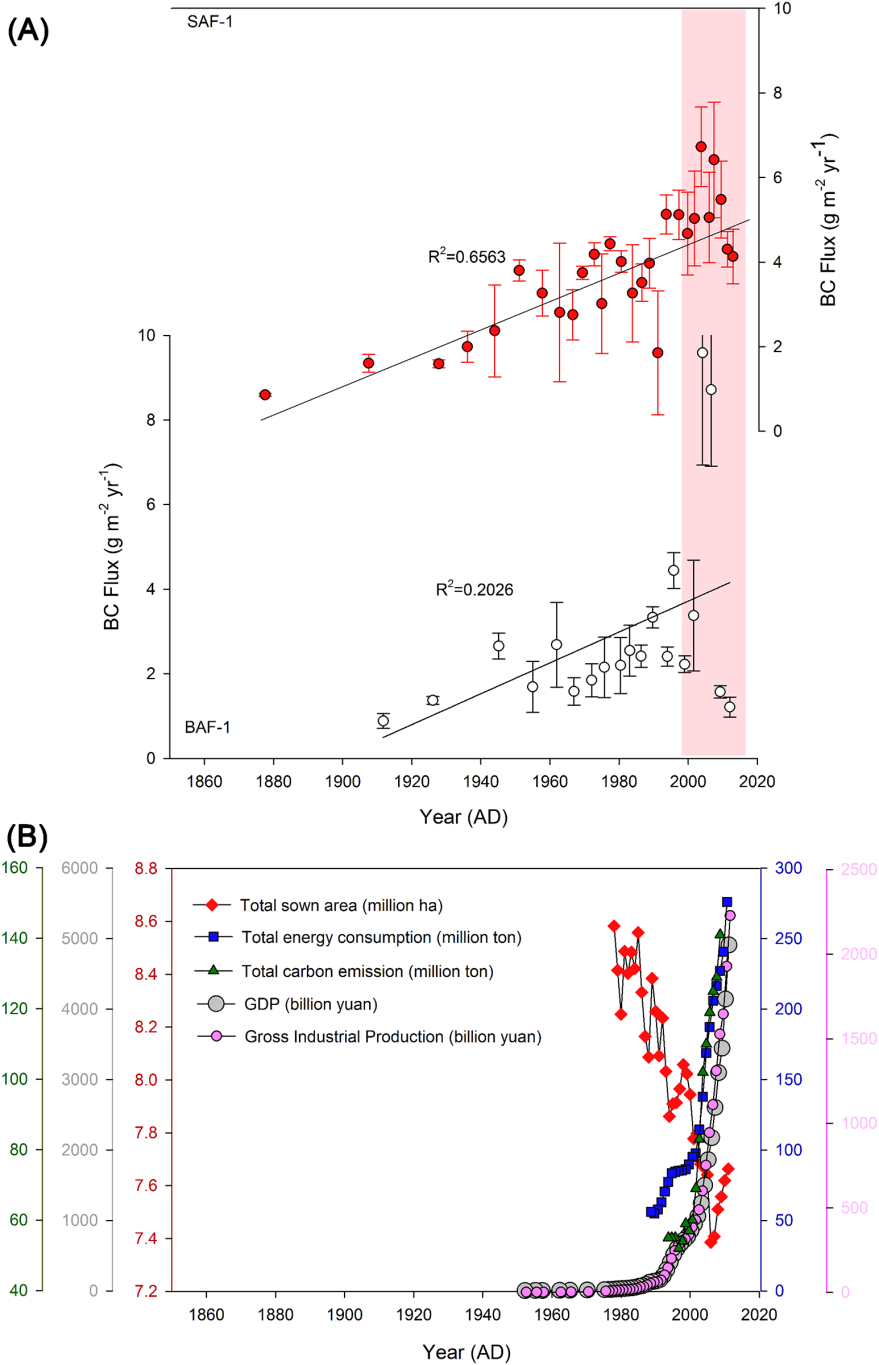
Historical trend of BC flux and its potential source. **(A)** Variation of BC flux from 1860 to 2013 reconstructed from two sediment cores (SAF-1 and BAF-1) in Yancheng coastal wetland, China. An obvious peaking period of BC flux is marked by red bar (last 20 years). Error bars represent the standard deviation (SD) of the mean of three parallel samples. **(B)** Variations of social and economic development indicators in Jiangsu province including gross domestic product (GDP), gross industrial production, total energy consumption and total sown area [49] and total carbon emission (1995–2010) [50].

As shown in Fig 7A, the temporal trend of BC fluxes to the Yancheng coastal wetlands shows a gradual and continuous increase (R^2^ = 0.6563 for SAF-1 and 0.2026 for BAF-1) and a significant peak in recent years (around 2000–2007). The history of BC fluxes can therefore be seen to follow the regional pollution history in Jiangsu province. We discount the possibility that the observed BC record reflects changes in local wildfire prevalence, given the location of the Yancheng coastal wetland within the developed region of China. Anthropogenic BC emissions from residential areas, industry, transportation and other sectors including power generation and agricultural waste burned in fields [47,48] are therefore likely the main controllers of BC concentration in the sampled sediments. We have used a number of metrics as broadly reflective of the development through time of these varied sources of BC. The gross domestic product (GDP), gross industrial production, total energy consumption and total sown area [49] and total carbon emission (1995–2010) [50] in Jiangsu province were used to assess the anthropogenic sources change. The increase in energy consumption and total carbon emission is accompanied by an increase of BC deposition in Yancheng coastal wetland and the rapid economic development over the last 20 years corresponds to the peak of BC flux (Fig 7B). Such a close relationship suggests changes in BC emitted from increasing human activities in the last 150 years control the BC flux change in this study. It is worth noting that the area of arable land is decreasing in Jiangsu province, which is an indicator of open biomass burning [19]. Given that the total sown area increased in the last seven years, it was more or less coincident with the BC flux decrease (Fig 7). This suggests that the biomass burning can not control the historical trend of BC emission and the contribution of agricultural waste burned in fields is smaller than the other anthropogenic sources associated with coal consumption. The small decline in BC flux over the past seven years agreed well with the patterns of SR and BC concentrations. At such surface layers, the intensive hydrological disturbance usually led to a lower accretion rate and BC concentration in sediments, which is possible to explain the small decrease in BC flux. Nevertheless, this would not invalidate the overall increasing trend of anthropogenic BC emission during the last 150 years at coastal Jiangsu, China.

## Conclusions

We studied two ^210^Pb-dated sediment cores from Yancheng coastal wetland in Jiangsu province of East China and reconstructed the historical trend of BC fluxes over the past 150 years, from the preindustrial to the modern period. Average BC contents are 0.51 mg g^-1^–0.69 mg g^-1^ and average BC fluxes are 2.94 g m^-2^ yr^-1^–3.79 g m^-2^ yr^-1^, which are comparable to those reported in other regions in the world. An increase in BC content and flux over time was observed, clearly reflecting increased emissions from anthropogenic activities. Higher BC concentrations in surface sediments and the maximum BC fluxes during the last 20 years coincide with the sharp rise in energy consumption and industrial emissions in recent decades. However, cost implications have limited our sampling effort to analyzing two cores in detail. Our data therefore present a preliminary pattern on the deposition of BC in coastal Jiangsu, which may guide future, more intensive analyses.
